# Frequency of twinning in southwest Nigeria

**DOI:** 10.4103/0971-6866.44104

**Published:** 2008

**Authors:** A. Akinboro, M. A. Azeez, A. A. Bakare

**Affiliations:** Department of Pure and Applied Biology, Ladoke Akintola University of Technology, Ogbomoso, Nigeria; 1 Cell Biology and Genetics Unit, Department of Zoology, University of Ibadan, Ibadan, Nigeria

**Keywords:** Diet, maternal influence, southwest Nigeria, twinning rate

## Abstract

**BACKGROUND:**

In the human species, twin is a type of multiple birth in which the mother gives birth to two offspring from the same pregnancy. The occurrence and frequency of twinning, however, varies across human populations. The maternal age, socio-environmental factors, increase in the use of contraceptives, the race of human population, increase in the spontaneous abortion rate, and seasonal variations are among the factors that could influence twinning rate. Information on twinning rates in southwest Nigeria is limited.

**AIMS:**

This study presents information on the frequency of twinning, as well as its analysis by maternal age, in four urban settings in southwest Nigeria. This is with the aim of extending current knowledge on the frequency of twinning in southwest Nigeria and contributing to the demographic studies in the country.

**MATERIALS AND METHODS:**

Data on single births and twin births from January 1995 to December 2004 were collected from the Oyo State General Hospital (OSGH), Wesley Guild Hospital (WGH), Obafemi Awolowo University Teaching Hospital (OAUTH), and Ekiti State Specialist Hospital (ESSH) in Ogbomoso, Ilesa, Ile-Ife, and Ado-Ekiti respectively. These were analyzed by year and maternal age groups of 15-19, 20-24, 25-29, 30-34, 35-39, 40-44, and 45-49 years according to the standard method.

**RESULTS:**

A frequency of twin births of 46.5 per 1000 deliveries and 46.2 per 1000 deliveries was recorded for Ilesa and Ile-Ife respectively. The frequency recorded for Ogbomoso and Ado-Ekiti was 38.5 and 22.1 per 1000 deliveries respectively. The overall average frequency of 40.2 per 1000 deliveries for the four hospitals ranks among the highest recorded rates of twin births in the world. The maternal age group of 25-29 years had the highest occurrence of twin births, while the lowest was recorded in the 45-49 years age group.

**CONCLUSION:**

This analysis reveals high incidence of twinning in the studied areas and supports previous assertion that the southwestern part of Nigeria has the highest twinning rate in the country and in the whole world. It is our opinion that diet, maternal history of twinning, and some socio-environmental factors may have influenced the results.

## Introduction

Twins in animal biology is a form of multiple births in which the mother gives birth to two offspring from the same pregnancy - some of the same gender, others of opposite. Giving birth to twins is a relatively rare event in humans, where occurrences vary considerably across populations.[1,2] The human female usually has a single baby in each pregnancy; but one in 90 pregnancies is a twin pregnancy; one in 8,100 pregnancies is a triplet pregnancy; and one in 729,000 pregnancies is a quadruplet pregnancy.[3] Alfonso *et al*.[4] reported that the rates of multiple births were the highest during the last three decades of the 18^th^ century, when the twinning rate was more than 17‰, the triplets rate was more than 3‰ and the quadruplet rate was almost 7 per 1 million births. In the whole world, analyses and estimation of multiple births have revealed approximately 125 million twins and triplets, out of which 10 million were just identical twins.[5]

Twins have been reported to be more frequent in African and Asian countries, and this is due to the higher proportion of fraternal twins; as identical twins occur equally often, whatever the race or age of the mother. Among Caucasians, fraternal twins occur more frequently in families with a history of twins, in older women, among women who have had several children previously, and after fertility treatment to induce ovulation.[3] Among the factors that could influence twinning are the maternal age and the race of human population,[1,6] socio-environmental factors, increase in the use of contraceptives, increase in the spontaneous abortion rate,[6–7] and seasonal variations.[8]

Frequency of twin birth varies considerably across human populations. Among the earliest reports of the incidence of twinning in Nigeria is that of Bulmer,[9] who reported an incidence of 44.9 twin births per 1000 births in Ibadan. Between 1967 and 1969, Nylander[10] reported twinning incidence of 48.3‰ and 57.2‰ for Ibadan and Igbo-ora respectively. Mosuro[11] obtained a higher incidence of twin births: 68.1‰ births for Igbo-ora, 37.2‰ births for Ibadan, and 26.9‰ births for Lagos. In Ilorin [12] and Calabar,[13] the incidence of twinning was 35.1‰ and 26.5‰ births respectively. In the northern part of Nigeria, the twinning rate was reported to be 39.7‰[14] and 28‰[15] births.

**Figure 1:**
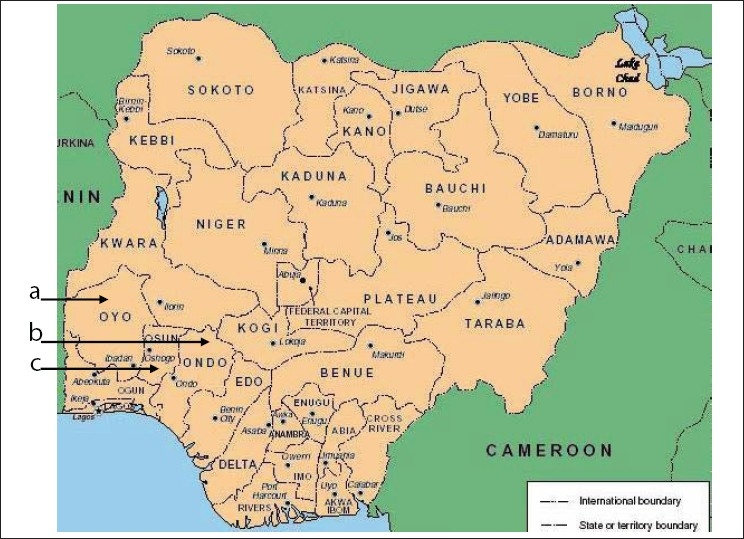
Map of Nigeria showing the states of the Federation. Data were collected from 3 states (arrowed) in Southwestern Nigeria, (a) Oyo state, (b) Ekiti state and (c) Osun state. Igbo-Ora in Oyo state (a), has the highest rate of twinning in the country and worldwide

Most reports on incidence of twinning in southwest Nigeria are from data obtained at Ibadan and Igbo-Ora in Oyo state and Lagos state.[9–11,16] These are just two out of the six states that constitute the southwestern part of Nigeria. In this study, we present information on the incidence of twinning in Ogbomoso, the second largest town after Ibadan in Oyo state [Figure 1]; Ile-Ife and Ilesa towns in Osun state [Figure 1]; and Ado-Ekiti, the capital of Ekiti state [Figure 1]. It is hoped that data obtained will extend current knowledge on the frequency of twinning in southwest Nigeria and contribute to the demographic studies in the country.

## Materials and Methods

The data used for this study were collected from the birth records of four different hospitals in three states of southwestern Nigeria [Figure 1]. These hospitals are Oyo State General Hospital (OSGH) at Ogbomoso in Oyo state; Wesley Guild Hospital (WGH) at Ilesa and Obafemi Awolowo University Teaching Hospital (OAUTH) at Ile-Ife in Osun state; and Ekiti State Specialist Hospital (ESSH) at Ado-Ekiti, Ekiti state. The data consists of single and twin births recorded for a period of 10 years, from January 1995 to December 2004. These were analyzed by year and maternal age group. Seven maternal age groups, viz., 15-19, 20-24, 25-29, 30-34, 35-39, 40-44, and 45-49 years, were considered for the possible influence of mother's age on twinning rate. All sets of data were not analyzed for type of twins delivered, i.e., whether monozygotic (identical) or dizygotic (fraternal), because of inadequate records. This is not expected to affect the results obtained in this study. The number of single births (*x*), the number of twin births (*y*), and the number of single births for every twin delivery (*x/y*) were determined. The number of twin births in every 1000 deliveries was computed as

$$\frac{\text{Twin deliveries }\left(y\right)}{\text{Total deliveries }\left(z\right)}\times 1000$$

## Results

Table 1 shows the annual incidence of twin births recorded in General Hospital, Ogbomoso, Oyo state, from 1995 to 2004. The highest twin births (53.8‰ births) were recorded in 2001, while the lowest twin births (26.8‰ births) were obtained in 2000. The average number of twin births pooled for the period was 38.5‰, and the average number of single births for each twin delivery was 24.9. Data analysis by maternal age shows that the highest incidence of twin births occurred in the 25-29 years age group, while the lowest was among the 15-19 years age group [Table 1]. No twin birth was recorded among the 45-49 years age group.

**Table 1:** Annual incidence of twin births recorded in Ogbomoso General Hospital, Oyo State, and analysis by maternal age for a period of 10 years (1995-2004)

Year	Single deliveries *(x)*	Twin deliveries *(y)*		Total deliveries *(z)*	Single for each twin delivery *(x/y)*		Twin births per 1000 deliveries
1995	483	15		498	32.2		30.1
1996	421	19		440	22.2		43.2
1997	411	17		428	24.2		39.7
1998	290	12		302	24.2		39.7
1999	289	15		304	19.3		49.3
2000	363	10		373	36.9		26.8
2001	334	19		353	17.6		53.8
2002	439	17		456	25.8		37.3
2003	497	19		516	26.2		36.8
2004	646	24		670	26.9		35.8
Total	4173	167		4340	24.9		38.5
**Maternal age group (yr)**							
Year	15-19	20-24	25-29	30-34	35-39	40-44	45-49
1995	2	2	4	2	1	4	-
1996	3	4	4	2	3	3	-
1997	2	3	4	2	3	3	-
1998	-	3	3	2	2	2	-
1999	2	3	2	3	2	3	-
2000	2	1	1	3	1	2	-
2001	3	3	4	2	3	4	-
2002	2	3	4	3	3	2	-
2003	2	3	5	4	2	3	-
2004	4	4	6	4	3	3	-
Total	22	29	37	27	23	29	-

The annual incidence of twin births recorded in Obafemi Awolowo University teaching hospital complex, Ile-Ife (OAUTH), is summarized in Table 2. The highest twin birth rate of 52.5‰ was recorded in 1995, and the lowest value of 41.2‰ was recorded both in 1997 and 2000. The average pooled value of 20.7 was obtained as the number of single births for each twin delivery. The average pooled result of twin births per 1000 deliveries over the period of ten years was 46.2‰. The maternal age range with the highest twinning incidence was 25-29 years, while the 45-49 years age group produced the lowest number of twin births over the period [Table 2].

**Table 2:** Summary of the annual incidence of twin births recorded in Obafemi Awolowo University teaching hospital complex, Ile-Ife, and analysis by maternal age for 10 years (1995-2004)

Year	Single deliveries *(x)*	Twin deliveries *(y)*		Total deliveries *(z)*	Single for each twin delivery *(x/y)*		Twin births per 1000 deliveries
1995	1678	93		1771	18.0		52.5
1996	1550	77		1627	20.1		47.3
1997	1560	67		1627	23.3		41.2
1998	1505	74		1579	20.3		46.9
1999	1257	61		1318	20.6		46.3
2000	1302	56		1358	23.3		41.2
2001	1080	50		1130	21.6		44.3
2002	940	49		989	19.2		49.6
2003	1331	65		1396	20.5		46.6
2004	1101	52		1153	21.2		45.1
Total	13304	644		13948	20.7		46.2
**Maternal age group (yr)**							
Year	15-19	20-24	25-29	30-34	35-39	40-44	45-49
1995	1	12	35	35	9	1	-
1996	1	16	34	16	9	1	-
1997	-	7	27	21	10	1	1
1998	-	9	31	19	12	3	-
1999	2	5	21	21	11	1	-
2000	2	8	17	14	13	2	-
2001	-	6	17	14	13	-	-
2002	-	7	18	14	9	1	-
2003	-	14	27	16	8	-	-
2004	-	7	25	13	5	2	-
Total	06	106	264	183	99	12	1

Table 3 presents the annual incidence of twin births recorded in Wesley Guild Hospital, Ilesa, Osun state. The variation in the incidence of twin births per 1000 deliveries over the years covered followed no specific pattern. The highest twin birth rate of 59.7‰ was recorded in 1998, while the lowest value of 32.7‰ was obtained in 1997. The average number of twin births per 1000 deliveries pooled was 46.5‰, indicating that 20.5 single births were recorded for each twin delivery. The age group 25-29 years had the highest record of 167 twin births (16.7 twin births per year), while the least number (0.3 twin birth per year) was recorded in the 15-19 years age group [Table 3].

**Table 3:** Summary of the annual incidence of twin births recorded in Wesley Guild Hospital, Ilesa, Osun state, and analysis by maternal age for a period of 10 years (1995-2004)

Year	Single deliveries *(x)*	Twin deliveries *(y)*		Total deliveries *(z)*	Single for each twin delivery *(x/y)*		Twin births per 1000 deliveries
1995	935	47		982	19.9		47.9
1996	884	42		926	21.0		45.4
1997	1007	34		1041	29.6		32.7
1998	1103	70		1173	15.8		59.7
1999	836	36		872	23.2		41.3
2000	850	40		890	21.3		44.9
2001	677	33		710	20.5		46.5
2002	541	28		569	19.3		49.2
2003	783	42		825	18.6		50.9
2004	603	29		632	20.8		48.1
Total	8219	401		8620	20.5		46.5
**Maternal age group (yr)**							
Year	15-19	20-24	25-29	30-34	35-39	40-44	45-49
1995	-	9	19	15	4	-	-
1996	-	9	18	8	3	3	1
1997	-	4	13	10	5	2	-
1998	-	10	29	13	16	2	-
1999	1	3	15	8	8	-	1
2000	2	8	12	9	7	-	2
2001	-	3	10	11	9	-	-
2002	-	4	15	6	2	1	-
2003	-	8	22	9	3	-	-
2004	-	3	14	8	3	1	-
Total	03	68	167	97	60	09	04

Table 4 shows the annual incidence of twin births recorded in State Specialist Hospital, Ado-Ekiti. The highest incidence of twin births per 1000 deliveries of 30.0‰ was recorded in 1998, while the lowest value of 11.8‰ was recorded in 1997. The pooled average number of twin births per 1000 deliveries was 22.1‰, indicating that 44.2 single births were recorded for each twin delivery. The highest number of twin births was recorded in the 30-34 years age group, while the lowest number of twin births was recorded in the age group 15-19 years. No twin birth was recorded among the 45-49 years age group [Table 4].

**Table 4:** Summary of the annual incidence of twin births recorded in State Specialist Hospital, Ado-Ekiti, Ekiti state, and analysis by maternal age for a period of 10 years (1995-2004)

Year	Single deliveries *(x)*	Twin deliveries *(y)*		Total deliveries *(z)*	Single for each twin delivery *(x/y)*		Twin births per 1000 deliveries
1995	464	7		471	66.3		14.9
1996	456	10		466	45.6		21.5
1997	504	6		510	84.0		11.8
1998	452	14		466	32.3		30.0
1999	575	12		587	47.9		20.4
2000	678	16		694	42.4		23.1
2001	680	17		697	40.0		24.4
2002	943	17		960	55.5		17.7
2003	1012	27		1039	37.5		26.0
2004	1050	28		1078	37.5		26.0
Total	6814	154		6968	44.2		22.1
**Maternal age group (yr)**							
Year	15-19	20-24	25-29	30-34	35-39	40-44	45-49
1995	-	-	2	3	2	-	-
1996	-	2	3	4	1	-	-
1997	-	1	3	2	-	-	-
1998	-	-	3	6	5	-	-
1999	-	-	3	5	3	1	-
2000	1	5	7	2	1	-	-
2001	-	3	6	5	3	-	-
2002	-	1	2	6	7	1	-
2003	1	4	8	4	10	1	-
2004	1	5	5	7	9	1	-
Total	03	21	42	44	41	04	-

**Figure 2:**
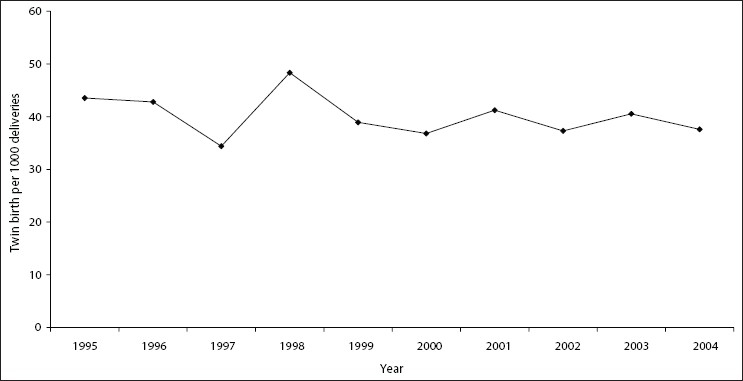
Annual incidence of twin births recorded in 4 hospitals in Southwest Nigeria between January 1995 and December 2004

**Figure 3:**
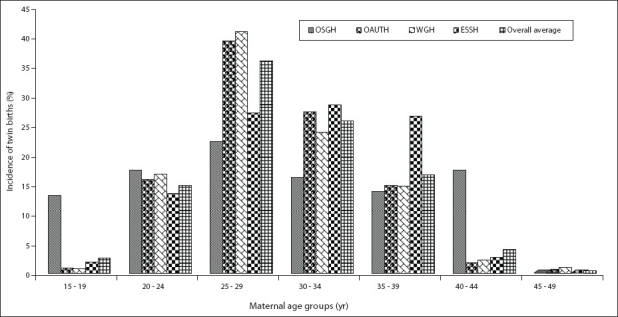
Incidence of twin births recorded in 4 hospitals in Southwest Nigeria between January 1995 and December 2004 analysed by maternal age OSGH: Oyo state General Hospital, Ogbomoso OAUTH: Obafemi Awolowo University Teaching Hospital, Ile-Ife in Osun state WGH: Wesley Guild Hospital, Ilesa in Osun state ESSH: Ekiti state specialist Hospital, Ado-Ekiti in Ekiti state Overall average: Average (%) incidence of twin births analyzed by maternal age in the 4 hospitals

Overall, 33,979 deliveries were recorded in the four hospitals between January 1995 and December 2004. The highest incidence of twin births occurred in 1998, while the lowest value of 34.4‰ was recorded in 1997 [Figure 2]. The average number of twin births in all the hospitals for the period of study was 40.2‰, while the average number of single births for each twin delivery was 23.9. The age group 25-29 years had the highest number of twin births (35.9%), while the lowest number recorded was in the age group 45-49 years [Figure 3].

## Discussion

In this study, the incidence of twinning in Ogbomoso, Oyo state; Ile-Ife and Ilesa, Osun state; and Ado-Ekiti, Ekiti state, all in southwest Nigeria, was considered. All the hospitals are government owned; and, in addition, OAUTH is a teaching hospital. In Nigeria, not all records of births are available in hospitals or birth registries as births occurring at home and births of unwanted or abandoned infants go unrecorded. Only 37.3% of births in Nigeria take place within health facilities.[17] Data collected for this study are assumed to be the rate at which twin births occurred in the places where the hospitals are located. There was approximately the same number of twin births (2‰ deliveries) at Ile-Ife and Ilesa town in Osun state; and their values of 46.5‰ and 46.2‰ were the highest in this study. These values were the double of what obtained for Ado-Ekiti and about 10‰ above the value recorded in Ogbomoso. With the exception of Ado-Ekiti, the incidences of twinning in the other three locations are higher than the 26.5 per 1000 births reported in Calabar;[13] 28 per 1000 in Jos;[15] 35.1 per 1000 in Ilorin;[12] and 39.7 per 1000 births for the Hausa population in northern Nigeria.[14] They are also higher than 11‰ twin births recorded in Kenya,[18] 20‰ twin births observed in Sub-Saharan Africa,[2] 16.1‰ twinning rate recorded in Nepal,[19] and 33.4/1000 and 26.6/1000 recorded for Accra and Kumasi in Ghana respectively.[20] The values in this study are higher than the African rate of 20‰ twin births[21] and rank among the highest twinning rates in the world.

Apart from genetic predisposition, a factor that may have also been influencing high twinning rate in southwest Nigeria is diet.[22] There is a general belief that the Yoruba's predisposition to high twinning rate is due to consumption of yam (*Discorea* sp.), which is believed to contain a natural hormone phytoestrogen, which may stimulate multiple ovulation.[23] Indigene of the studied areas are known to have preference for food prepared in different forms from yam. Maternal history of twinning (though not considered in this study) might also have contributed to the high incidence of twinning observed in this study and previous studies from southwest Nigeria. This is because the probability of a subsequent twin pregnancy is increased fourfold in mothers of twins, and the risk of having dizygotic (DZ) twins is roughly double for a woman whose mother or sister has DZ twins.[24]

The incidence of twinning among the races of the world has been extensively studied. Worldwide changes have occurred in the pattern of twinning rates in recent decades. The highest twinning rate is observed to occur among the Negroids, while the lowest occurred among the Mongoloid population.[1] In Nigeria, the incidence of twinning was estimated to be 45‰.[7] Bulmer[9] recorded 44.9‰ incidence of twinning in Ibadan, while 48.3‰ and 57.2‰ incidences of twin births were respectively obtained for Igbo-Ora and Ibadan between 1967 and 1969.[10] On an average, the highest record of twinning is among the Yorubas of southwest Nigeria, with approximately 50-53 twin births per 1000 deliveries.[25] These rates were suggested then as the highest in the world. Between 1985 and 1989, 68.1‰ twin births were recorded for Igbo-Ora, while 37.2‰ and 26.9‰ twin births were recorded for Ibadan and Lagos respectively.[11]

The values obtained in this study, especially those of Ogbomoso and Ado-Ekiti, are lower than the overall estimated average for the Yorubas of southwest Nigeria.[10,25] This may mean that there is a decline in the rate of twinning in southwest Nigeria, an observation that is in concordance with the observations of Mosuro.[11] The studied locations are urban towns inhabited by the Yorubas. But with migration, other ethnic groups are also found living in these areas. This might have led to interbreeding resulting in admixture of genes and hence reduction in twinning rates. In general, twinning rates appear to be modified by both migration and inter-ethnic mixing.[26]

Another factor that may have influenced the twinning rate in the present study is social class. Nylander[6] reported that the twinning rate in the lowest social class was over twice that in the middle and highest social class in Nigeria. This was particularly true of Igbo-Ora, Nigeria,[10] which was and still is a rural settlement that is believed to be homogenous culturally, socially, ethnically, and may even be consanguineous; and it is having the highest incidence of twin births in Nigeria, as well as in the world. Ogbomoso, Ilesa, Ile-Ife, and Ado-Ekiti are towns with middle and high social classes in Nigeria, hence the decline in the twinning rate. Changing societal values such as reduction in polygamy, family planning policy on reduction in the number of children produced per family, and more women pursuing career goals have had a resultant effect of decrease in twinning rate.

In some studies in other countries, the decline in twinning rate has been related to an increase in industrialization and urbanization[27] and population density,[28] resulting in “psycho-social stress.”[29] The high population growth rate, immigration from the rural areas into the towns, the gradual breakdown of family structure, and the continuing urbanization of areas that were traditionally agricultural may have all produced an environment in which psycho-social stress is a significant factor. It seems possible, therefore, that psycho-social stress factors may be responsible, at least in part, for the decline in twinning rate in the studied locations. It is not possible to substantiate this theory with the data available from the present study. However, the fact that previous studies from southwest Nigeria[11,16] showed a possible continuing decline in incidence of twinning in urban settings in southwest Nigeria, coupled with the belief that psycho-social stress factors in the studied areas might have increased over this period in terms of a continuing increase in population density and political strife, does not contradict the theory.

In addition, a history of oral contraceptive use has been suggested to contribute to the decline in twinning by acting directly to reduce the probability of double-ovulation.[30] The decline may also be influenced by a decrease in the probability of conception. It seems, therefore, that no single factor can explain the trends observed. It is increasingly evident that the explanation of the variability of twinning rates is very complex and is not just of interest *per se* but may disclose new insights into the role of genetic versus environmental factors in the reproduction of man. This seemingly declining rate of the incidence of twinning is not peculiar to Nigeria. Several studies of populations elsewhere in the world have shown a decline in twinning rate during the 20^th^ century.[7,29,31,32]

The maternal age group that had the highest twin birth rate in Ilesa, Ile-Ife, and Ogbomoso was the 25-29 years age group; while at Ado-Ekiti, it was the 30-34 years age group. The 25-29 years age group of mothers with highest twin births obtained for Ilesa, Ile-Ife, and Ogbomoso is in concordance with the records obtained in Igbo-Ora, Ibadan, and Lagos;[11] Jos[15] and Calabar.[13] This appears to be the age group with the highest record of twin births in southwest Nigeria. According to Mosuro,[11] the relative youthfulness of the age group with the highest twinning rate in the present study may be related to modern trend in Nigerian women toward earlier marriage and having fewer children, particularly in urban areas. As a result of such social changes, there are fewer older mothers now having children. This translates to a lowering of birth rate generally, and consequently, a downward trend in the twinning rate in the studied locations. The data of Ado-Ekiti for the 30-34 years age group is in accord with data obtained from Europe and White United States populations,[1] and is still within the general belief that the rate of twinning appears to increase with maternal age reaching a peak at about 37 years.[1] This may be the reason for the lowest twinning rates in the 15-19 years age group since women in this age group are most likely to have their first delivery. Also, women within the age group 46-49 years in most cases are perimenopausal or may have completed their families, hence the lower incidence of twin birth in this age group.

This study has presented data on twin births in four major towns of southwestern Nigeria. Analysis of data reveals high incidence of twinning in these towns and supports the previous assertion that the southwestern part of Nigeria has the highest twinning rate in the country and in the whole world. Further studies are needed on the possible influence of diet, environment, and societal changes on rates of twinning in Nigeria.
